# Swimmer, Protect Thyself: Cleaning Up the Pool Environment

**DOI:** 10.1289/ehp.118-a476

**Published:** 2010-11

**Authors:** Angela Spivey

**Affiliations:** **Angela Spivey** writes from North Carolina about science, medicine, and higher education. She has written for *EHP* since 2001 and is a member of the National Association of Science Writers

In 2006 a 6-year-old Nebraska boy was treated in a hospital intensive care unit for respiratory distress caused by chemical inflammation and swelling of his airways after spending three hours swimming in an indoor motel pool. Twenty-four other people who visited the pool in the same time period experienced milder eye and respiratory irritation.

The pool was closed the next day. Inspectors found a broken ventilation system and a water level of chloramine—a respiratory and ocular irritant that forms when chlorine disinfectant combines with sweat, urine, or other organic matter^1^—that was eight times the state maximum. The free chlorine level in the pool was less than half the state minimum. The person who maintained the pool had no verifiable training in pool management and safety.^2^

This was no isolated incident. Two years later an analysis of data from more than 111,000 routine pool inspections in 13 states by the Centers for Disease Control and Prevention (CDC) showed that 12.1% of pools were immediately closed upon inspection for serious health or safety violations.^3^

As these examples demonstrate, the United States needs to do a better job of maintaining its public swimming pools. That task will involve standardizing pool codes, which currently are regulated at the state and local level and vary widely. Such an effort is under way, with the CDC sponsoring development of a science-based Model Aquatic Health Code (MAHC) that will serve as a guide.^4^

But part of the responsibility lies with swimmers, who can reduce their own risks and make the pool healthier and safer for others by remembering they’re really taking a communal bath and behaving accordingly. A new national model pool code can help, but swimmers also have to protect themselves.

## No Directions Included

Why isn’t there federal regulation of swimming pools? The U.S. Environmental Protection Agency monitors nature areas used for recreation (lakes, rivers, and oceans), but since pools are manmade, there’s no federal authority with jurisdiction. That means pool codes vary by state or, in some cases, by county.

Many state and local pool codes do not require pool operators to have any training in safety, disinfection, or chemical handling, even though, as both common sense and science suggest, such training likely leads to safer pools. A 2007 study conducted by health officials in Nebraska—where at the time of the study only two counties required operator training of nonmunicipal public pools such as those at hotels—found that violations in disinfectant level and pH were twice as likely at pools in counties that did not require operator training.^5^

Tracynda Davis, director of environmental health programs at the nonprofit National Swimming Pool Foundation, has seen firsthand how states can end up with codes that may not be ideal. When she worked for the Wisconsin Department of Health, she tried to change the state pool code to require operator training for all public pools, but she met resistance as soon as she formed an advisory board. “The Innkeeper Association, made up of people who run hotels and motels, came to the table and said, ‘We are not going to agree with that, and if you take it to the legislature, we will oppose it.’ They found such a requirement too onerous and costly,” Davis says.

The law Davis ultimately helped pass still stands in Wisconsin today.^6^ Under that code, only water attractions or water parks, including water slides, are required to have a trained operator; traditional pools, spas, and hot tubs are not. In some other states, operator training is required only for municipal pools, but hotel and apartment complex pools are commonly excluded from that requirement.

Making changes to state or local codes can take years. In Wisconsin there is not a state board of health to approve rules promulgated by the Department of Health, so pool regulations must go through the state legislature. Davis worked on the code revisions in Wisconsin for six years—four to hold advisory committee meetings, write the code, and put it out for public comment, then two to get the resulting legislation approved.

Such protracted processes are not uncommon because code changes often are one of numerous tasks on the to-do list of environmental health officials spread too thin, Davis says. For example, at the same time she was revising the pool code in Wisconsin, she also was working on regulation of food inspections and of tattoo and piercing establishments.

“The challenge for public health officials at the state level is that they may not have time to do adequate research,” Davis says. “They want to do a good job, but they may not have a lot of experience with new technology that industry brings, such as ultraviolet light disinfection.” So when members of advisory committees differ on, say, the minimum chemical disinfectant that should be required in combination with ultraviolet treatment, a public health official needs science to make informed decisions.

## A Code for Health

Such challenges could be eased by the national MAHC currently under development.^4^ Initially spurred by a rising incidence of illnesses associated with recreational waters, such as cryptosporidiosis (“crypto”), the CDC in 2005 began bringing together representatives from federal, state, and local government, academia, and the pool and water park industry to create the code, which state and local governments will be encouraged to adopt.

“We think a national effort to bring all these people together to develop a more standardized code that can be updated regularly will help bring uniformity to codes across the country. It will prevent state and local health departments from having to reinvent the wheel every time,” says Michael Beach, associate director for healthy water at the CDC and a member of the MAHC steering committee.

However, adoption of the code will not be mandatory. “The CDC is not a regulatory authority,” Beach says. “If we put things in the code that a state or local health department doesn’t think they can ever pass in a legislative body or they can’t regulate or that they don’t like, then it’s not going to be adopted, and we’ve all wasted our time.”

The code will address all aspects of swimming pool design, construction, operation, and maintenance that could affect safety and public health. Eleven technical committees are drafting sections of the code covering disinfection and water quality, facility design and construction, facility maintenance and operation, hygiene facilities, lifeguarding and bather supervision, monitoring and testing, operator training, recirculation systems and filtration, regulatory program administration, risk management and safety, and ventilation and air quality. A twelfth committee will summarize existing data on pool contamination burden and advise the other committees.

It’s a big undertaking—the code’s outline alone spans 14 pages. Originally the CDC had projected that all modules would be up for public comment by July 2008, but the majority are not yet ready for public comment. The steering committee is striving to have all the modules out for comment by the end of 2010, Beach says. Each individual module will be available for public comment on the MAHC website^4^ for 60 days.

After each module is revised to address comments, the entire code will be put up for a final 60-day comment period. Once the code is completed, the plan is to update it every two years in much the same way the Food and Drug Administration’s Food Code^7^ undergoes updating.

Appendices, or annexes, to the code that are not adoptable as regulation or law will provide a scientific basis for the code and rationale for best practices, says Michele Hlavsa, chief of the CDC Healthy Swimming Program. Among these annexes is a guide for standardization of inspection data with recommendations such as using separate data fields for each inspection violation and using a unique identifier for each pool facility and for each body of water at the facility (the kiddie pool, the adult pool, etc.). Beach says such improved recordkeeping can help state and local health departments focus limited inspection resources on, for instance, pools that have a repeated pattern of violations.

## Learning from Experience

The increase in recreational water illnesses such as cryptosporidiosis that spurred the MAHC effort was partly due to large outbreaks in several states. That year, the number of reported cases of cryptosporidiosis, a diarrheal illness caused by the chlorine-resistant protozoan *Cryptosporidium*, almost doubled in the United States, from 6,479 cases in 2006 to 11,657 in 2007.^8^

Utah, in particular, experienced one of the largest cryptosporidiosis outbreaks in U.S. history. The state Department of Health confirmed more than 1,902 cases, nearly 100 of which involved hospitalization; in previous years, the state had reported an average of 14.8 cases per year. Much of the evidence associated the outbreak with swimming in contaminated pools—80% of the patients reported swimming exposure at just 450 different pools. And 20% of the patients reported they had swum in a public pool while ill with diarrhea.^9^

After the first eight cases were confirmed, state public health officials implemented measures to contain the outbreak, such as advising pool operators to post notices asking swimmers with diarrhea in the past two weeks to refrain from swimming. Operators also were advised to institute weekly hyperchlorination^10^ to kill *Cryptosporidium*, which can survive for weeks in the normally recommended amount of chlorine. But with 12 autonomous local health departments, each with its own unique political pressures, some of the departments did not get the message or did not follow these measures on first notice.

After the outbreak Utah changed its code to include a prearranged, formalized plan for containing outbreaks when a cryptosporidiosis watch or warning is issued, giving the state more authority to take emergency measures such as hyperchlorination or restricting swimming during an outbreak. They also implemented other prevention measures, such as mandating that all pools log the same types of data about fecal accidents and the pool management’s response, forbidding changing diapers at poolside, and requiring that all pool restrooms include diaper-changing stations.

Retrospective analysis of the Utah outbreak showed that using the state’s usual surveillance methods, which relied on cases confirmed by a doctor’s diagnosis, left a considerable lag between when cases began and when the state was notified. For instance, when the state’s surveillance was showing 6–8 reported cases, the actual number of cases totaled 50.^11^ This happens with many types of outbreaks, and some of the discrepancy is due to the inevitable lag time between when people first feel sick and when they go to a doctor.

But Utah has instituted new surveillance measures to detect cryptosporidiosis earlier. These include a weekly look at data from an independent laboratory that does most of the testing for cryptosporidiosis in the state, including the number of tests ordered and the proportion that are positive. These data are available earlier than case numbers, which are confirmed only after individual doctors receive test results, make a diagnosis, and notify the health department of positive results.

“When we looked retrospectively, it turned out there was a big spike in the amount of testing and the positivity rate that actually would have notified us of the outbreak before the reported cases,” says Robert Rolfs, director of the Division of Disease Control and Prevention at the Utah Department of Health.

Rolfs adds that a national model code would have been helpful both in guiding reaction to the outbreak and in making code changes afterward. “I think it’s a really good idea to have a model rule because it’s nice when you’re making regulations to have something to fall back on,” he says. “We had to make a lot of decisions without necessarily having a very clear, strong science case for what would work.”

## Disinfection By-Products

The chlorine level recommended for swimming pools by the CDC is 1–3 ppm, and the recommended level for bromine is 2–5 ppm.^12^ Most germs are actually killed at the lower ends of these ranges, but extra disinfectant often is needed to accommodate the organic matter—such as urine, sweat, and dirt—that is brought in by swimmers. That’s because all the contaminants and compounds on an unshowered body react with the free disinfectant in the pool, reducing the amount available to kill pathogens, Hlavsa says.

This same reaction also forms highly volatile disinfection by-products (DBPs), many of which are respiratory irritants. A study reported in this issue of *EHP* identified more than 100 DBPs, some not previously found, in a sample chlorinated pool.^13^ DBPs have been studied for decades,^14^ but this was the first study to characterize all the DBPs in a single pool.^15^

Some studies have suggested an increased risk of asthma from exposure to DBPs even in well-maintained pools, especially indoor pools. For instance, several studies conducted by Belgian researchers suggested DBPs as a trigger for asthma, especially among young children. One such study of 430 children showed an association between swimming pool attendance before the age of 2 years and increased incidence of bronchiolitis (inflammation of the small air passages) and, later in childhood, increased risk of asthma.^16^

But these studies were not conclusive, and subsequent work has not been able to replicate this finding.^17^ Most recently, Laia Font-Ribera of the Centre for Research in Environmental Epidemiology (CREAL), Barcelona, and colleagues reported that, among 8,750 children aged 7–8 years, more than half of whom swam more than once a week, swimming was not associated with increased risk for any of the evaluated symptoms overall.^18^

On the contrary, children who swam had lower prevalence of asthma and use of asthma medication and better lung function at age 7 in the CREAL study.^18^ The authors note that confounding by factors such as a “healthy swimmer effect”—that is, children who are uncomfortable swimming because of respiratory symptoms are less likely to do so regularly—cannot be ruled out.

One reason for the differences in findings may be that the measures of asthma available in retrospective studies do not match current criteria for medical diagnosis of asthma. David Callahan, a physician who leads the asthma epidemiology research team at the CDC, points out that some studies that found a link involved children under age 5 years, whose wheezing bouts may not have been true asthma but rather caused by colds that overwhelmed their smaller airways. “Really well-controlled studies have not been done,” Callahan says.

Other studies have linked swimming pool exposure to increased bladder cancer risk, and a paper published in this issue of *EHP* shows short-term changes in two biomarkers of genotoxicity (DNA damage that may lead to cancer) in people who swam for 40 minutes in a chlorinated pool.^19^ “These are only biomarkers, not cancer itself,” says lead author Manolis Kogevinas, an epidemiologist at CREAL. “But in principle you don’t want to have things messing up your DNA.”

If further studies validate an increased cancer risk associated with swimming pool exposure, measures to reduce the amount of chemicals used to disinfect swimming pool water may be needed, Kogevinas says.

## The Role of the Swimmer

The large outbreaks in Utah and elsewhere have highlighted the need for better public education of swimmers. “Many of these outbreaks likely begin with diarrheal contamination at a pool. That means someone was in the pool who shouldn’t have been in the first place,” Beach says.

As scientists explore the possible links between microbes, chemicals, and health, some say the known risks can be mitigated by improved swimmer hygiene, which includes showering with soap before entering the pool. This not only reduces the chances of pools becoming contaminated with *Cryptosporidium* and other germs but also can help reduce the amount of chemicals needed in the pool.

“If swimmers reduce the load to the pool—sweat, urine, and fecal matter—by activities such as showering before swimming and taking frequent bathroom breaks, this should help to reduce the amount of chemicals needed to achieve proper pool disinfection,” says Judy LaKind, an associate professor at the University of Maryland School of Medicine and president of LaKind Associates, who consults to government and industry.

Beach agrees. “People don’t like to hear it, but one of the key issues here, beyond what we can do in operations and inspections, is that the swimming public has to learn that this is a communal bathing area, and we need to up the ante on hygiene,” he says.

Beach also would like to see swimmers “vote with their feet” and leave pools that aren’t properly maintained. He goes so far as to advise swimmers to test the pool chemicals themselves with portable test trips (see “Protect Yourself from the Health Risks of Swimming,” p. A480).

Overall, environmental health experts say that, especially with obesity rampant, the benefits of swimming far outweigh the known risks. In the United States, swimming is the third most popular sport, with more than 52 million people swimming at least six times per year.^20^

The exact number of illnesses and accidents associated with swimming each year is unknown because it’s uncertain how many incidents go unreported. CDC surveillance shows that reported cases of cryptosporidiosis and giardiasis, another gastrointestinal illness associated with recreational water, numbered 31,451 in 2007,^8^,^21^ compared with more than 314 million reported swimmer visits.^20^

“We think the risk is low considering how frequent the exposure is,” Beach says. “Swimming is a great activity, and we want to continue to see people out there exercising and having fun, but we want to eliminate some of the downsides as well. We can do that through prevention—through code and educating the public.”

## Do Your Part to Keep the Pool Clean and Safe

Contaminants that swimmers bring into the pool can make other swimmers sick. They also combine with the chemicals used to clean pools, which not only reduces the amount of disinfectant that’s free to kill germs but also forms new compounds with known adverse health effects.

- Shower with soap before entering the water.
- Take children on frequent bathroom breaks, and check their diapers often.
- Change diapers in the bathroom, not at the poolside.
- Wash children thoroughly (especially their bottoms) with soap and water after they use the toilet or their diapers are changed and before they enter the water.
- Protect others by not swimming if you are experiencing diarrhea. This is essential for children in diapers.
- If you are diagnosed with crypto sporidiosis, do not swim for at least two weeks after diarrhea stops.
- Don’t urinate in the pool. In a 2009 online survey of 1,000 Americans, 17% of respondents admitted doing this.^22^

## Protect Yourself from the Health Risks of Swimming

- Don’t swallow pool water or get it in your mouth.
- A well-maintained pool (even an indoor pool) should have little to no disinfectant odor and should not cause swimmers to cough or their eyes to water.
- Pool sides should not feel slimy or sticky.
- Ask the management how often the pool is tested; the disinfectant level and pH should be tested twice a day.
- Swimmers can bring their own test strips to check the water for proper chlorination and pH levels. These strips can be purchased at most home improvement and pool supply stores or online for about $10.00 for 50 strips. Follow manufacturers’ instructions. Free chlorine levels should be 1–3 ppm, or bromine level should be 2–5 ppm. The pH level should be 7.2–7.8. At a pH above 7.8, chlorine will not effectively kill germs. At a pH below 7.2, chlorine will actually be more effective, but swimmers might experience skin or eye irritation.
- If you notice any of these problems, or if your test shows the pool isn’t properly disinfected, notify the management, and don’t swim in the pool until the problem is corrected.
- Many people with asthma find swimming to be a beneficial exercise. But if asthma symptoms worsen on days you swim, disinfection by-products may be a trigger for you. Change pools or talk with the management about proper pool maintenance to reduce respiratory irritants.

## Figures and Tables

**Figure:**
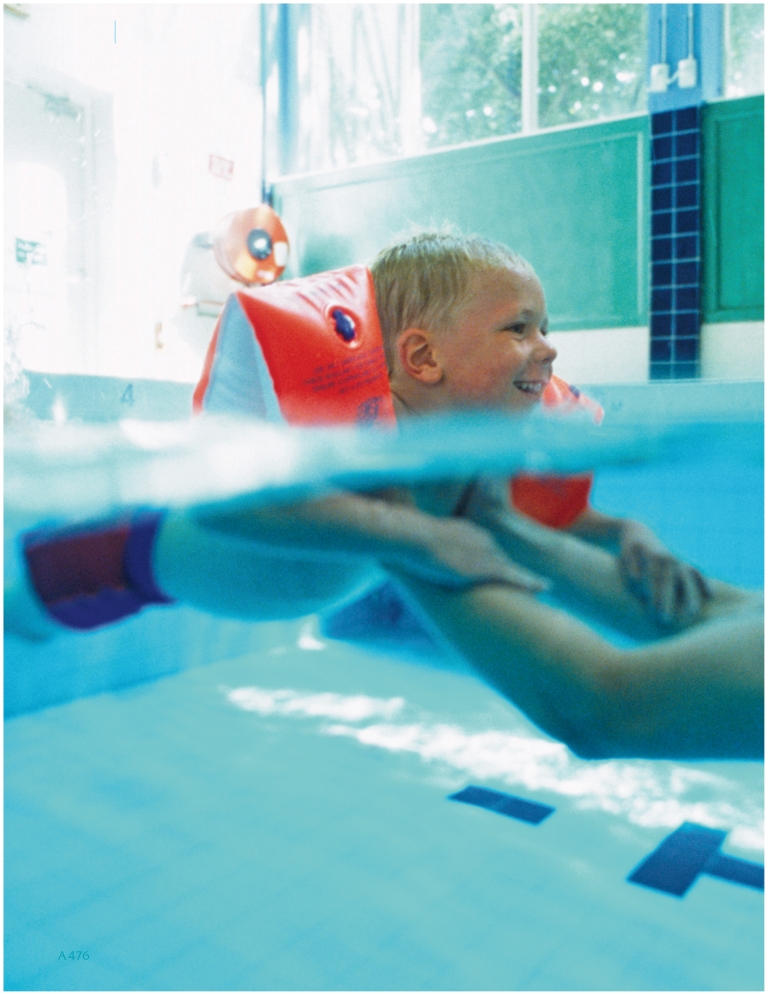


**Figure:**
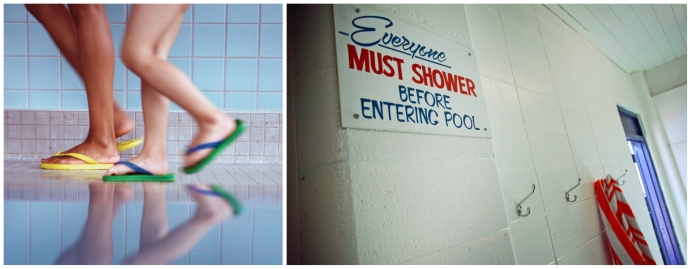


**Figure:**
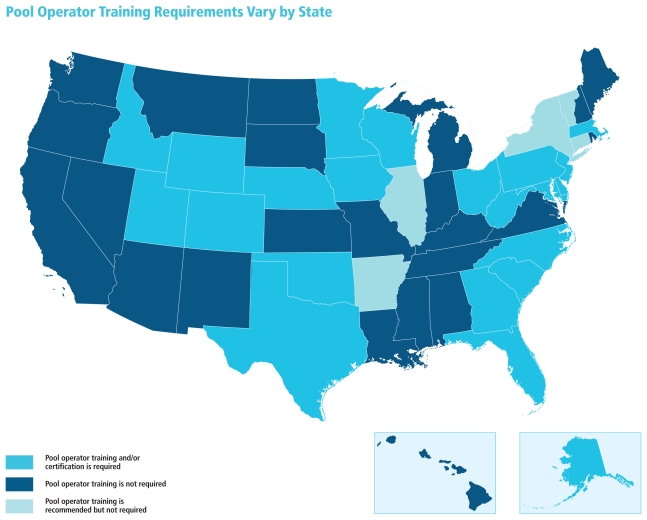
States are about equally divided between those that require training or certification for pool operators and those that do not. Five states encourage training or require simply that operators be “qualified,” “knowledgeable,” or “familiar with equipment” without specifying parameters of this qualification. In some instances (for instance, Jefferson County, Alabama; Suffolk County, New York), local regulations mandate more stringent training requirements than state code does.

**Figure:**
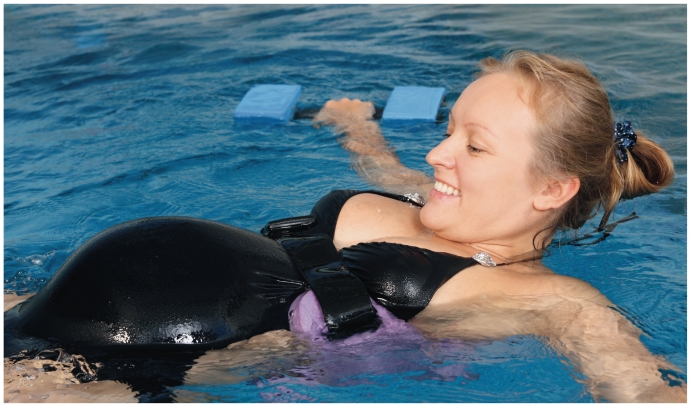


**Figure:**
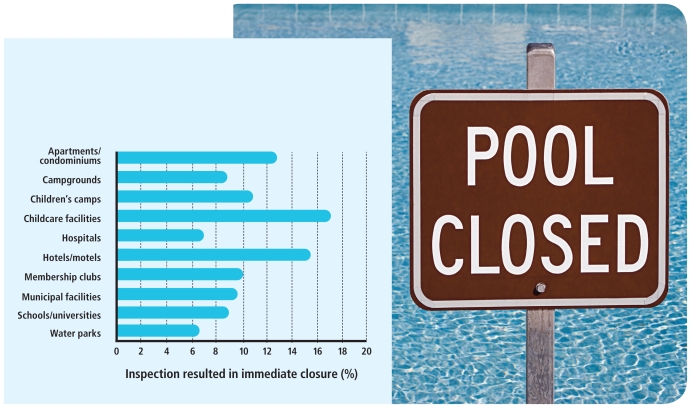
Pools Closed The CDC analyzed 2008 data for more than 111,000 routine pool inspections in 13 states and found that, overall, 12.1% of inspections resulted in immediate closure due to serious health violations.^3^ Some settings were more likely than others to have closures. For instance, childcare facilities led the group with 17.2% of inspections resulting in immediate closure. Water parks—which in some states are subject to more stringent operator training rules than other types of pool facilities—had the fewest at 6.4%.
